# The Scientific Basis of Eugenics

**Published:** 1912-10

**Authors:** 


### Department of Eugenics.

Conducted by Dr. Malone Duggan and Dr. Theo. Y. Hull.

### THE TEXAS STATE SOCIETY OF SOCIAL HYGIENE.

Headquarters: San Antonio, Texas.

DR. MALONE DUGGAN, President, San Antonio.
DR. F. E. DANIEL, 1st Vice President, Austin.
PROF. A. CASWELL ELLIS, 2d Vice President, Austin.
MRS. ELI HERTZBERG, 3d Vice President, San Antonio.
DR. THEO. Y. HULL, Secretary, San Antonio.
MR. W. L. EVANS, Treasurer, San Antonio.

EXECUTIVE COMMITTEE.

| | | |
|---|---|---|
| Dr. Malone Duggan, Chm. | Hon. J. C. Willacy. | Dr. W. A. King. |
| Dr. Theo. Y. Hull, Sec'y. | Mr. W. L. Evans. | Dr. J. W. Torbett. |
| Dr. W. F. McCaleb. | Dr. Frank D. Boyd. | Dr. F. C. Walsh. |
| Hon. C. H. Jenkins. | Dr. J. R. Nichols. | Dr. Joe Reuss. |
| Rev. A. W. S. Garden. | Dr. J. M. McCutchan. | Dr. Frank Paschal. |

### The Scientific Basis of Eugenics.

In the last analysis, love may be said to be the basic principle of eugenics, for it alone inspires the desire for reproduction, and is, for that reason, the primordial motive in life. But the modus operandi by which race culture may be affected is based on the laws of heredity. Environment, as an element in race culture, is a sociological problem, and, while it has its influence in the betterment of mankind, it is limited to immediate conditions, and does not affect the inherent element concerned. Therefore, we must rely upon heredity, mainly, and the laws governing the same, for our success in this movement.

Heredity is based on the continuity of the germ-plasm, which exists in the male and female alike, and is resident in the germ-cells. They are separate and apart from the *soma,* body cells, and are not influenced by any modification of them. The only way by which they may be modified is through the action of taxemias, bacteremias, intoxications and disturbed metabolic states sufficiently active to effect the general bodily fluids from which the germ-plasm receives its nutrition. (Herein lies the reason for personal hygiene as a prophylaxis in race culture.)

Reproduction has to do only with the germ-plasm, which, after it forms the individual, takes up its abode in the reproductive organs ready to continue the cycle. The germ-cells are called *gametes* or marrying cells. They contain all of the mental and physical factors out of which the child is developed. When they fuse to form the new being, they are called *zygotes*, and the life thus formed is double in all its qualities, partaking of all the qualities contained in both parent cells. The qualities in both cells may be the same or different. When they are the same with reference to some particular character, they are pure-bred as to that character. When dissimilar, the resulting organism is cross-bred. Therefore, the individual may inherit two similar or two dissimilar portions, or he may have the same ingredient from each absent, or he may receive only one ingredient from each.

Taking these laws as a basis, Mendel has shown how by segregation and dominance specific character can be formed. As stated by Barker, "If we could know the unit characteristics which both parents possess as well as those which both lack, and also the unit characters in which they differ, and if we further know for each characteristic whether it was due to a presence or an absence, we could go far towards predicting the result of a particular marriage." Thus we are on the threshold of deliberately planning characters of health, probity, courage, and honor, or weed out characters of weakness, dishonesty, cruelty, immorality, etc. But there is this difficulty, while man corresponds biologically to the laws of the vegetable and animal kingdom, he is not governed so much by natural selection as they are when let alone, but he has the power of interrupting these laws at will. He can not change a natural law, but he can and does apply them to meet his personal ends. Eugenics would utilize the laws of heredity as its working basis and invoking environments to create sentiment and co-operation to direct these laws for the accomplishment of the end sought.

